# Biological Activities of Alkaloid Fraction of *Fritillaria karelinii* (Fisch. ex D. Don)

**DOI:** 10.5812/ijpr-165081

**Published:** 2025-11-11

**Authors:** Ardak Omarbekova, Sebastian John Adams, Mamdouh Nabil Samy, Ubaidilla Datkhayev, Lashyn Kiyekbayeva, Elmira Kapsalyamova, Bayan Sagindykova, Serzhan Mombekov, Samir Anis Ross

**Affiliations:** 1 School of Pharmacy, Asfendiyarov Kazakh National Medical University, Almaty, Kazakhstan; 2 National Center for Natural Products Research, School of Pharmacy, The University of Mississippi, Oxford, USA; 3 Department of Drug Technology, South Kazakhstan Medical Academy, Shymkent 160019, Kazakhstan; 4 Department of Pharmacognosy, Faculty of Pharmacy, Cairo University, Cairo, Egypt; 5 School of Pharmacy, Asfendiyarov Kazakh National Medical University, Almaty, Kazakhstan; 6 Department of Drug Technology, South Kazakhstan Medical Academy, Shymkent, Kazakhstan; 7 Department of BioMolecular Sciences, Division of Pharmacognosy, School of Pharmacy, The University of Mississippi, Oxford, USA

**Keywords:** *Fritillaria karelinii*, Kazakhstan, Liliaceae, HPTLC, GC/MS, Alkaloids

## Abstract

**Background:**

*Fritillaria karelinii* (Fisch. ex D. Don) belongs to the lily family (Liliaceae) and grows in Central Asia, Iran, Pakistan, and the Xinjiang Autonomous Region of China. The underground bulbs of this plant are traditionally used as both food and medicine, especially within the framework of traditional Chinese medicine (TCM). Although earlier studies reported promising antioxidant activity, comprehensive investigations combining morpho-anatomical, phytochemical [high-performance thin-layer chromatography (HPTLC) and gas chromatography-mass spectrometry (GC-MS)], and biological analyses are lacking. This study addresses this gap by providing an integrative characterization of *F. karelinii* bulbs.

**Objectives:**

The present study aims at a comprehensive characterization of the bulbs of *F. karelinii*, including their morphological, anatomical, and chemical features. The chemical profile of the samples was studied using HPTLC and GC-MS. Additionally, the complete extract was analyzed for antioxidant, antimicrobial, and cytotoxic activity to assess its biological potential.

**Methods:**

Histochemical analysis was performed to localize alkaloids and other metabolites in the parenchyma cells of *F. karelinii* bulbs. To obtain a characteristic chromatographic profile and identify the main alkaloids, HPTLC was applied as a fingerprinting tool. The GC-MS was used primarily for profiling fatty acids and other volatile/semi-volatile compounds in the methanolic extract, while acknowledging that this method does not capture polar metabolites such as phenolics or flavonoids. The biological activity of the samples was evaluated through free radical scavenging (DPPH), ferric reducing antioxidant power (FRAP), antimicrobial assays, and cytotoxicity testing.

**Results:**

Histochemical analysis confirmed the presence of alkaloids in the parenchyma cells of the bulbs, which also contained abundant starch granules. The HPTLC revealed a distinct fingerprint, highlighting the major alkaloids. The GC-MS profiling of the methanolic extract detected 16 fatty acids and other volatile components, with linoleic acid (40.86%) and palmitic acid (30.58%) as the dominant fatty acids, followed by linolenic acid (13.30%) and stearic acid (5.85%). The alkaloid-rich fraction contained 5α-cevan-3β,20-diol (19.65%) and fritillarin (18.86%) as prominent alkaloids. Their identification was confirmed by comparison with the National Institute of Standards and Technology (NIST) library, which showed high similarity scores [match factor (MF) = 890, reverse match factor (RMF) = 905 for 5α-cevane-3β, 20-diol; MF = 875, RMF = 900 for fritillarin]. The crude extract showed no detectable DPPH scavenging, antimicrobial, or cytotoxic activities under the tested conditions. In contrast, it demonstrated moderate ferric reducing capacity (1.0578), though lower than the reference standard gallic acid (1.8705).

**Conclusions:**

The bulbs of *F. karelinii* were shown to contain diverse classes of secondary metabolites, as confirmed by histochemical analysis. The HPTLC and GC-MS profiling identified 5α-cevan-3β,20-diol and fritillarin as the predominant steroidal alkaloids, whose RMF and MF values confirm their structural reliability. However, under the tested experimental conditions, the methanolic extract did not exhibit significant antioxidant, antimicrobial, or cytotoxic activities. These findings indicate that while the species possesses a complex phytochemical profile, further investigations are required to clarify its pharmacological relevance. The present results highlight the need for more comprehensive studies, including the evaluation of other compound classes (e.g., phenolics, flavonoids, alkaloids) and optimized experimental conditions, before firm conclusions regarding its medicinal potential and traditional uses can be drawn.

## 1. Background

Genus *Fritillaria* L. from the lily family (Liliaceae) is mainly distributed in temperate zones of the Northern hemisphere. Representatives of the genus are found in regions such as Central Asia (including the territories of modern Kazakhstan, Kyrgyzstan, Tajikistan, Uzbekistan, and Turkmenistan), as well as in Iran, Pakistan, and the Xinjiang Autonomous Region of the People's Republic of China (1, 2). According to World Flora Online, this genus currently comprises 148 species (3). Historically, it was classified under the genus *Rhinopetalum* (Fisch. ex D. Don) Sweet in the Flora of the USSR (4) and Conspectus Florae Asiae Mediae (5), but later reclassified under the name *Fritillaria* (6, 7). In Kazakhstan, six species are recognized within this genus. One of them, *Fritillaria karelinii* (Fisch. ex D. Don) Baker (previously known as *Rhinopetalum* Fisch. ex Sweet), is distributed across Northern Kazakhstan, Turkmenistan, Iran, and Xinjiang (northwestern China). Initially, this species was often confused with *Fritillaria* *gibbosa* Boiss. and even classified alongside *Fritillaria stenanthera* Regel (8-10). However, taxonomic studies, based on floral petal characteristics, helped distinguish it as a separate species (8).

The underground parts, particularly the bulbs, of various *Fritillaria* species have been widely used as both food and medicine. Certain species' roasted bulbs have historically been consumed by Native American communities as a food source (11). In traditional medicine, *Fritillaria* is used to treat a wide range of diseases such as dyspepsia, chest injuries, tuberculosis, cough, asthma, gout, bronchitis, dysuria, sinusitis, furunculosis, stomatitis, malaria, and anemia. In addition, the plant is used to strengthen the immune system, as well as for childhood exhaustion, fever, burning, tuberculosis intoxication, bronchial asthma, cardiovascular pathologies, and respiratory and nervous system disorders (1). *Fritillaria* species are known to contain a diverse array of secondary metabolites, with alkaloids being the predominant group found in their bulbs, along with terpenoids, steroidal saponins, and phenylethanoids (10). Various pharmacological effects of dried *Fritillaria* bulbs have been investigated, including antitussive, expectorant, antiasthmatic, antineoplastic, anti-inflammatory, antihypertensive, anti-cholinesterase, antibacterial, antiviral, antioxidant, antinociceptive, anti-allergic, neuroprotective, and antidiabetic activities (12).

In traditional Chinese medicine (TCM), *F. karelinii* is referred to as Sha-Beimu (13) and has traditionally been used to manage respiratory conditions such as cough, expectoration, and asthma. Previous phytochemical investigations of this species have led to the identification of various isosteroidal alkaloids, including karelinine, 5-epikarelinine, 27-epiebeienine, ebeienine, persicanidine B, heilonine, and fritillarine (14, 15). Additionally, the ethyl acetate fraction derived from *F. karelinii* bulbs has demonstrated promising antioxidant activity (16). Despite its medicinal significance, the anatomical characteristics of this plant remain underexplored, with only one study focusing on the morpho-anatomy of *F. karelinii* bulbs (17). Recent research has emphasized the importance of anatomical and morphological traits in the identification of medicinal plants (18, 19). Botanical and microscopic studies are critical for ensuring accurate species authentication. Since differentiating between species based solely on floral structures and tubers can be challenging, microscopy plays an essential role in verifying internal structural parameters for proper identification. Light microscopy (LM) has been employed for detailed histological and histochemical analysis (20). Furthermore, phytochemical analysis using high-performance thin-layer chromatography (HPTLC) is an essential tool for assessing histochemical profiles (21). In addition, gas chromatography-mass spectrometry (GC-MS), one of the most efficient and widely used techniques for profiling major secondary metabolites — including lipophilic, volatile, and derivatized hydrophilic constituents — was utilized to determine the chemical composition of *F. karelinii* bulbs (22-24).

The utilization of plant-derived natural products for the treatment of microbial infections presents advantages over synthetic drugs due to their relatively lower incidence of side effects. Research on their pharmacological, toxicological, and antimicrobial activities has drawn significant interest due to their potential effectiveness against various infectious agents (25). Thus, the present study aimed to analyze the morpho-anatomical characteristics of *F. karelinii* bulbs, establish their histochemical and HPTLC alkaloid profiles, and conduct a detailed GC-MS analysis, along with evaluating their antioxidant, antimicrobial, and cytotoxic properties.

## 2. Objectives

The aim of this study was to determine the morphological, anatomical, and chemical profiles of *F. karelinii* bulbs using HPTLC and GC-MS. Additionally, the total extract was analyzed for antioxidant, antimicrobial, and cytotoxic activities.

## 3. Methods

### 3.1. Plant Material

The bulbs of authentic *F. karelinii* were collected in April 2021 at a temperature of 22 - 24°C from the South Kazakhstan region, Kazygurt district, coordinates N42°03'19.11" E69°40'31.83". They were dried in a hot air oven at 100°C for 6 hours and then for an additional 36 hours at room temperature. The plant sample received the NCNPR registration number and was placed in the collection of the BR at the NC for the Study of Natural Compounds at the University of Mississippi.

### 3.2. Macro and Microscopic Analysis

The external morphology of the plant was observed using a NIKON SMZ-U stereomicroscope (Japan), with images captured by a NIKON DS-Fi1 camera connected to the microscope via NIS-Element BR software. Fresh hand sections of the tubers were used for the analysis. Transverse sections were stained with toluidine blue O for histological observations. Histochemical evaluations were performed using stains designed to localize starch and alkaloids (26, 27). Photomicrographs were obtained using an Olympus BX53 fluorescence microscope with a DP74 camera system and CellSens Standard version imaging software (Olympus Corp., Tokyo, Japan).

### 3.3. High-Performance Thin-Layer Chromatography Analysis

The air-dried bulbs (500 g) were macerated with methanol (500 mL, repeated 3 times, each for 24 hours) at room temperature. After combining the extracts, the solvent was evaporated under reduced pressure at 35 - 40°C, resulting in 10.0 g of dried residue. The methanol extract was fractionated using VLC with silica gel in a column (500 g, 0.063 - 0.200 mm; Merck, Darmstadt, Germany) and eluted with a gradient system of n-hexane, DCM, ethyl acetate, and methanol, yielding 4 fractions. These fractions were grouped based on their chemical similarity and monitored using thin-layer chromatography, then concentrated using a rotary evaporator. The resulting fractions were labeled RK-M1 (0.037 g), RK-M2 (0.059 g), RK-M3 (0.111 g), and RK-M4 (0.193 g).

### 3.4. Instrumentation and Chromatographic Conditions for High-Performance Thin-Layer Chromatography

The HPTLC analysis was performed using silica gel plates 60 F254 (20 × 10 cm, 200 microns, Millipore Sigma, USA) and a CAMAG system (Switzerland), including a Linomat 5 applicator (100 µL), an automatic ADC2 development chamber, a DigiStore2 scanner, and winCATS software (v1.4.3) and visionCATS (v2.0). The samples were applied in strips 8 mm wide at a distance of 10 mm from the bottom edge at a rate of 5 µL/s. Mobile phase: CHCL_3_–MeOH–H_2_O–FA (17:6:1:1). The chamber was pre-saturated with 25 mL of eluent at 23 ± 2°C and a relative humidity of 39 ± 5%. After development, the plates were treated with vanillin-sulfuric acid reagent using an immersion device (0.5 cm/s) and heated at 100°C for 3 minutes. Visualization was performed at UV 254 and 366 nm, and after treatment with Dragendorf reagent — at 366 nm and in visible light.

### 3.5. Gas Chromatography-Mass Spectrometry Analysis

The GC-MS analysis was performed on an Agilent 7890A GC system coupled to an Agilent 5975C mass spectrometer using a DB-1MS capillary column (60 m × 0.25 mm, 100% dimethylpolysiloxane). The carrier gas is helium (1 mL/min). Temperature program: 50°C (1 min) → increase by 5°C/min to 280°C, shutter speed 10 min. The injector is at 280°C, the separation mode is 50:1; the interface temperature is 280°C. The MS operated in the electronic ionization mode (70 eV); the source temperature was 230°C, and the quadrupole temperature was 150°C; mass scanning: 40 - 500 m/z, after 7-min exposure. Connection identification is performed using the National Institute of Standards and Technology (NIST) library (v2.3).

### 3.6. Determination of Fatty Acids Content

The methanol extract was analyzed for its fatty acid content using the GC-MS method. Twenty milligrams of extract was dissolved in 20 mL of CH_3_OH, refluxed with 2 mL of H_2_SO_4_ for 10 hours (28).

### 3.7. Evaluation of Antioxidant Activity

#### 3.7.1. DPPH Free Radical Scavenging Assay

The ability of the *F. karelinii* bulb extract to scavenge free radicals was measured using the DPPH assay, with gallic acid used as a positive control. A 3 mL solution of 6 × 10^-5^ M DPPH radicals was mixed with 0.1 mL of the test solution at concentrations of 0.25, 0.5, 0.75, and 1.0 mg/mL. The solutions were stored in the dark for 30 minutes, then optical densities were measured at 520 nm. The DPPH inhibition percentage was calculated using the formula: DPPH inhibition (%) = $\frac{A0\;-\;At}{A0\;\times \;100}$, where A0 is the optical density of the control sample, and At is the optical density of the working sample (29, 30).

#### 3.7.2. Ferric Reducing Antioxidant Power Assay

The total antioxidant capacity of the plant samples was evaluated using the ferric reducing antioxidant power (FRAP) technique. To the reaction mixture, 0.25 mL of 0.2 M phosphate buffer (pH = 6.6) and 0.25 mL of 1% potassium hexacyanoferrate (III) solution were added. The mixture was incubated for 20 minutes at 50°C, then 0.25 mL of 10% trichloroacetic acid was added to stop the reaction. After centrifugation, the top layer was mixed with distilled water and 0.1% FeCl_3_. The optical density was measured at 700 nm. Results were compared with gallic acid. Samples were tested in triplicate at concentrations of 0.25, 0.5, 0.75, and 1.0 mg/mL at a temperature of 20 ± 2°C (29, 30).

### 3.8. Evaluation of Antimicrobial Activity

The antimicrobial activity of the complete extract and its fractions was re-evaluated against a number of pathogenic microorganisms, including yeast and mold fungi (*C. albicans*, *C. neoformans*, *A. fumigatus*), as well as bacterial strains: Methicillin-resistant *S. aureus* (MRSA), *E. coli*, *P. aeruginosa*, *K. pneumoniae*, and vancomycin-resistant *enterococci* (VRE). Susceptibility tests were performed using a modified version of the CLSI methodology. A final DMSO concentration of 1% was used, and serial dilutions were made in 20% DMSO/saline. The optical density was measured at 530 nm or fluorescence at 544ex/590 em after incubation (31).

### 3.9. Evaluation of Cytotoxic Activity

The cytotoxic activity was assessed using the brine shrimp lethality assay with *Artemia salina *larvae, a widely recognized and cost-effective preliminary toxicity screen. Acute lethality was determined by comparing the number of dead larvae in treated samples with those in the negative control (artificial seawater without test substances). Actinomycin D served as a positive control, producing significant mortality (63 - 96%) across the tested concentrations. Larvae were exposed to three concentrations of the methanolic extract of *F. karelinii* bulbs (10, 5, and 1 mg/mL). Under these experimental conditions, the extract did not demonstrate significant cytotoxic activity, with only 4% mortality observed at the highest concentration tested. These findings suggest either that the bioactive compounds are present in very low amounts in the crude extract, or that different extraction approaches, fractionation, or broader concentration gradients may be required to reveal potential cytotoxic effects. It is important to note that while the *A. salina* lethality assay provides valuable preliminary insights into acute toxicity, it cannot be directly extrapolated to mammalian or human systems. Moreover, the relatively high concentrations tested (1 - 10 mg/mL) may not reflect pharmacologically relevant levels, and potential issues of solubility or bioavailability in the assay medium could have influenced the outcomes. Therefore, the absence of observed toxicity in this model should be interpreted with caution. Future studies will include cytotoxicity testing on mammalian cell lines, expanded concentration ranges, and fractionated extracts, alongside appropriate statistical analyses, to provide a more comprehensive evaluation of the cytotoxic potential of *F. karelinii* bulbs.

## 4. Results

### 4.1. Macro- and Micro-scopic Characteristics of Fritillaria karelinii Bulbs

**Figure 1.**
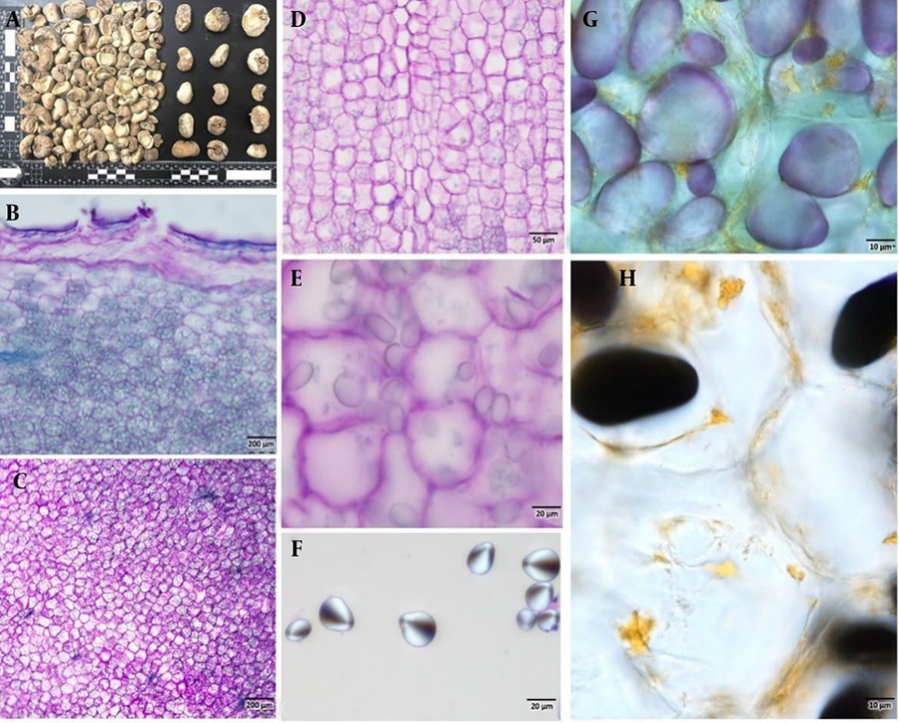
A, external morphology of *Fritillaria karelinii* bulbs; B and C, transverse section; D, longitudinal section of bulbs stained with toluidine blue O, highlighting the internal structure composed of parenchymatous cells densely packed with starch grains; E – H, close-up view of starch grains; G and H, histochemical staining to identify starch grains and alkaloids in the cells; dark blackish-blue staining indicates the presence of starch grains, while yellow-orange coloration confirms the presence of alkaloids (scale bar: B and C = 200 μm, D = 50 μm, E and F = 20 μm, G and H = 10 μm).

The bulb measures approximately 2 - 3 cm in diameter (Figure 1A) and lacks an outer scaly covering. It has a pale whitish color, a soft texture, and consists of 3 - 5 cloves. When dried, the outer surface becomes hard, while the fractured ends remain brittle. Microscopic examination revealed that the outer cork layer is 2 - 4 cells thick, often compressed into a single dense layer (Figures 1B - D). The parenchymatous ground tissue is densely packed with abundant starch grains, which exhibit ovoid to elliptical shapes (Figures 1E - H). The central region of the bulb contains scattered vascular traces. Histochemical analysis confirmed the presence of alkaloids localized around the cell walls (Figures 1G and H).

### 4.2. High-Performance Thin-Layer Chromatography Analysis

**Figure 2.**
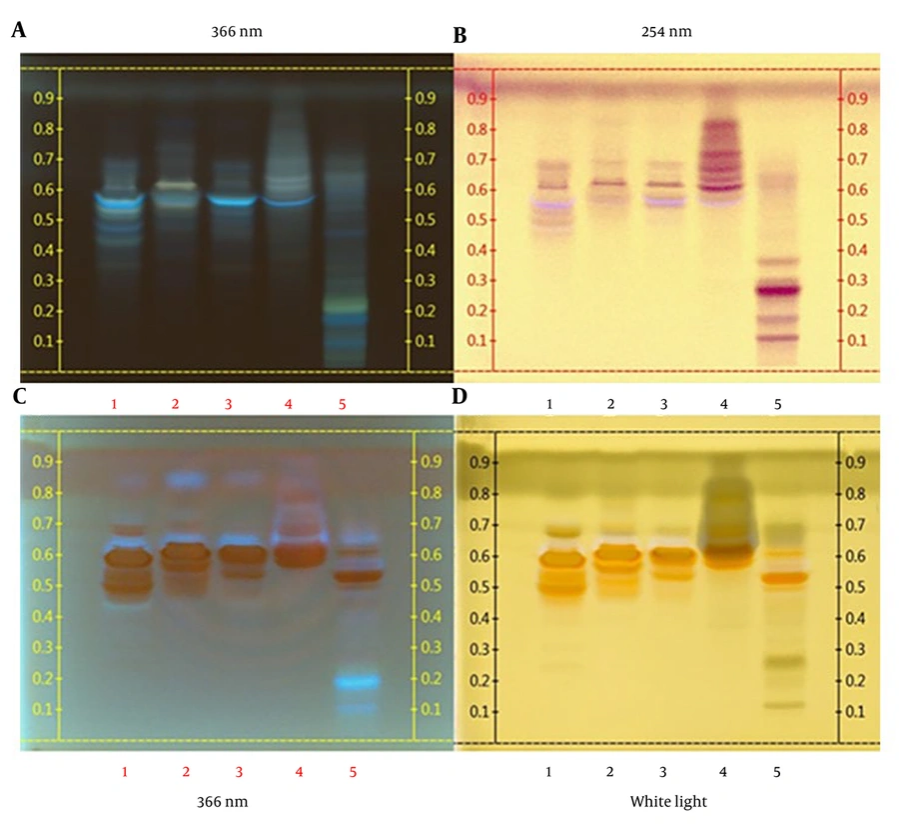
High-performance thin-layer chromatography (HPTLC) fingerprint profiles of the methanol extract and fractions of the bulb extracts: A, without derivatization; B, derivatized with vanillin-sulfuric acid; and C and D, derivatized with Dragendorff’s reagent [the tracks correspond to: RK-M1 (1), RK-M2 (2), RK-M3 (3), RK-M4 (4), and crude extract (5)].

The HPTLC fingerprinting of secondary metabolites in the crude bulb extract and purified fractions of *F. karelinii* was conducted, revealing the presence of alkaloids, flavonoids, polysaccharides, saponins, and terpenoids. After derivatization with vanillin-sulfuric acid reagent, these compounds were detected, as illustrated in Figures 2A and B. The presence of alkaloids was confirmed by the appearance of orange bands in the chromatogram after derivatization with Dragendorff’s reagent, observed under UV 366 nm and white light (Figure 2C and D). The methanolic crude extract of the bulbs exhibited eight bands, with Rf values in increasing order: 0.10, 0.20, 0.30, 0.46, 0.50, 0.62, 0.75, and 0.85. The optimal separation was achieved using a mobile phase composed of CHCl_3_-MeOH-H_2_O-FA in a ratio of 17:6:1:1.

### 4.3. Gas Chromatography-Mass Spectrometry Analysis

#### 4.3.1. Gas Chromatography-Mass Spectrometry of Fatty Acids

Fatty acid methyl esters were synthesized using the methanol-sulfuric acid esterification method, as described by Krishnamurthy et al. (32). The GC-MS analysis of the fatty acid fraction revealed the presence of 16 fatty acid methyl esters (Table 1). The main components identified were methyl linoleate (40.86%) and methyl palmitate (30.58%), followed by methyl linolenate (13.30%), methyl stearate (5.85%), 16-octadecenoic acid, methyl ester (2.70%), docosanoic acid, methyl ester (2.52%), tetracosanoic acid, methyl ester (1.03%), and heptadecenoic acid, methyl ester (1.01%).

**Table 1.** Gas Chromatography-Mass Spectrometry of Fatty Acids of the Bulbs of *Fritillaria karelinii*

No.	Retention Time (min)	Compound Name	Molecular Weight	Molecular Formula	Area%
**1**	26.8	Tridecanoic acid, 12-methyl-, methyl ester	242.3975	C_15_H_30_O_2_	0.59
**2**	28.3	7-Hexadecenoic acid, methyl ester, (Z)-	268.4348	C_17_H_32_O_2_	0.7
**3**	28.9	Pentadecanoic acid, methyl ester	256.4241	C_16_H_32_O_2_	0.84
**4**	30.5	9-Hexadecenoic acid, methyl ester, (Z)-	268.4348	C_17_H_32_O_2_	1.28
**5**	30.9	Hexadecanoic acid, methyl ester (methyl palmitate)	270.4507	C_17_H_34_O_2_	30.58
**6**	32.8	Heptadecenoic acid, methyl ester	284.4772	C_18_H_36_O_2_	1.01
**7**	34.1	9,12- Octadecanoic acid (Z,Z)-, methyl ester (methyl linoleate)	294.4721	C_19_H_34_O_2_	40.86
**8**	34.2	9,12,15-Octadecatrienoic acid, methyl ester, (Z,Z,Z) (methyl linolenate)	292.4562	C_19_H_32_O_2_	13.30
**9**	34.3	16- Octadecenoic acid, methyl ester	296.4970	C_19_H_36_O_2_	2.70
**10**	34.6	Octadecanoic acid, methyl ester (Methyl stearate)	298.5038	C_19_H_38_O_2_	5.85
**11**	35.3	9,12-Octadecadienoic acid (Z,Z)-, ethyl ester (linoleic acid ethyl ester)	308.4986	C_20_H_36_O_2_	1.26
**12**	36.4	Nonadecanoic acid, methyl ester	312.5304	C_20_H_40_O_2_	0.31
**13**	38.1	Eicosanoic acid, methyl ester	326.5570	C_21_H_42_O_2_	0.87
**14**	41.3	Docosanoic acid, methyl ester	354.6101	C_23_H_46_O_2_	2.52
**15**	42.8	Tricosanoic acid, methyl ester	368.6367	C_24_H_48_O_2_	0.67
**16**	44.2	Tetracosanoic acid, methyl ester	382.6633	C_25_H_50_O_2_	1.03

#### 4.3.2. Gas Chromatography-Mass Spectrometry of Alkaloidal Fraction

The GC-MS chromatogram of the alkaloidal fraction showed two predominant alkaloids: 5α-cevane-3β, 20-diol (19.65%) and fritillarine (18.86%) in the bulbs of *F. karelinii* (Table 2). 

**Table 2.** Gas Chromatography-Mass Spectrometry of Alkaloid Fraction of *Fritillaria karelinii*

No.	Retention Time (min)	Compound Name	Molecular Weight	Molecular Formula	Area%
**1**	26.8	5α-cevane-3β, 20-diol	415.6620	C_27_H_45_NO_2_	19.65
**2**	28.3	3β,20-dihydroxy-5α -cevan-6-one (fritillarine)	429.6450	C_27_H_43_NO_3_	18.86

#### 4.3.3. Identification of Alkaloids by Gas Chromatography-Mass Spectrometry Analysis

**Figure 3.**
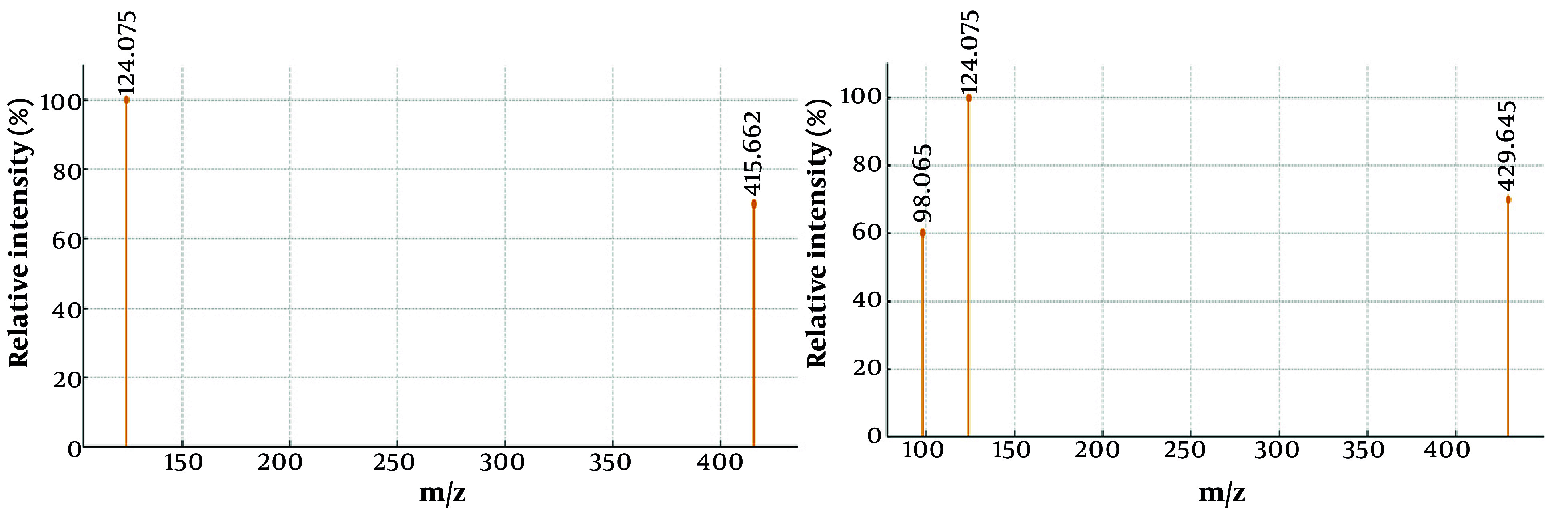
Mass spectra of A, 5α-cevane-3β,20-diol; and B, fritillarine

**Figure 4.**
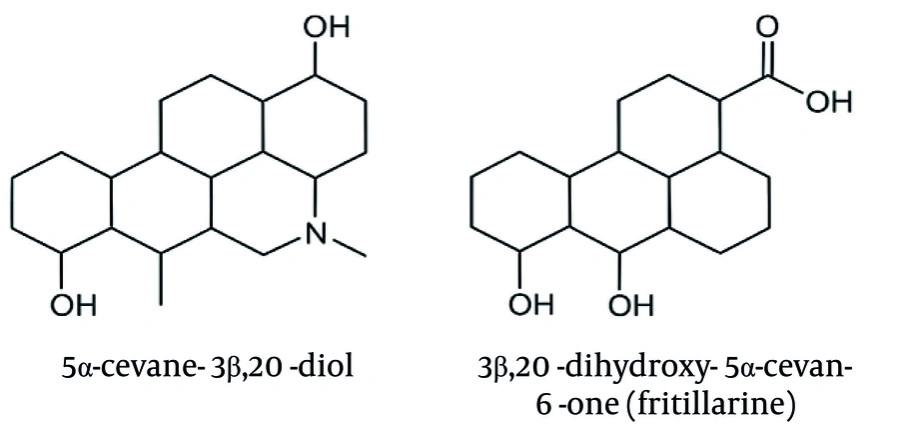
Structural formulas of the main alkaloids identified in the bulbs of *Fritillaria karelinii*: 5α-cevane-3β,20-diol (19.65%) and fritillarine (18.86%)

The GC-MS analysis of the alkaloid fraction revealed the presence of two major steroidal alkaloids belonging to the cevanine type. Compound identification was accomplished by comparing the obtained mass spectra with reference spectra from the NIST library. The similarity between the experimental and reference spectra was assessed using match factor (MF) and reverse match factor (RMF) values, confirming the high reliability of compound identification (Table 3). The representative mass spectra of the identified alkaloids, 5α-cevane-3β,20-diol and fritillarine, are shown in Figures 3A and B, while their corresponding structural formulas are presented in Figure 4. 

**Table 3.** Gas Chromatography-Mass Spectrometry Characteristics of the Identified Alkaloids

No.	Retention Time (min)	Compound	Molecular Formula	Molecular Weight (Da)	Area (%)	MF	RMF	Major Fragment Ions (m/z, Relative Intensity, %)
**1**	26.8	5α-Cevane-3β,20-diol	C_27_H_45_NO_2_	415.6620	19.65	890	905	415 [M]^+^ (12%), 400 (18%), 382 (26%), 125 (100%), and 97 (45%)
**2**	28.3	3β,20-Dihydroxy-5α-cevan-6-one (fritillarine)	C_27_H_43_NO_3_	429.6450	18.86	875	900	429 [M]^+^ (10%), 414 (21%), 396 (24%), 125 (100%), and 98 (42%)

Abbreviations: MF, match factor; RMF, reverse match factor.

The GC-MS analysis of the alkaloid fraction revealed two main steroidal alkaloids of the cevanine type, identified as 5α-cevane-3β,20-diol and 3β,20-dihydroxy-5α-cevane-6-one (fritillarin), with retention times of 26.8 and 28.3 minutes, respectively. Their identification was confirmed by comparison with the NIST library, which showed high similarity scores (MF = 890, RMF = 905 for 5α-cevane-3β,20-diol; MF = 875, RMF = 900 for fritillarin, Table 3). High MFs, along with characteristic fragment ions (m/z 415, 400, 382 for the first compound and 429, 414, 396 for the second), confirmed the accurate identification of both cevanine-type alkaloids. The representative mass spectra of 5α-cevane-3β,20-diol and fritillarin are shown in Figure 3, while their corresponding structural formulas are presented in Figure 4. These findings indicate that 5α-cevane-3β,20-diol and fritillarin are the predominant steroidal alkaloids in the bulbs of *F. karelinii*, contributing to its characteristic chemical profile.

### 4.4. Antioxidant Activity

#### 4.4.1. DPPH Free Radical Scavenging Activity

The total extract demonstrated weak 2,2-diphenyl-1-picrylhydrazyl (DPPH) free radical scavenging activity (30.52%), in contrast to the standard compound gallic acid (93.40% DPPH inhibition) at the tested concentration of 1 mg/mL.

#### 4.4.2. Ferric Reducing Antioxidant Power Method

The total extract was assessed for its total antioxidant capacity using the FRAP assay, which measures the ability of antioxidant molecules to reduce a ferric tripyridyltriazine (Fe^3+^-TPTZ) complex to its ferrous form (Fe^2+^-TPTZ) at low pH. The extract exhibited significant ferric reducing potential (1.0578 µg/mL gallic acid/g dry weight). In comparison, the reference compound gallic acid showed the highest reducing power (1.8705 µg/mL gallic acid/g dry weight).

### 4.5. Antimicrobial Activity

The antimicrobial activity of the methanol extract and various fractions of *F. karelinii* bulbs was evaluated using the method described by Elkhouly et al. (24). However, the extracts did not show any inhibition of the growth of *C. albicans*, *C. neoformans*, *A. fumigatus*, MRSA, *E. coli*, *P. aeruginosa*, *K. pneumoniae*, or VRE at concentrations ranging from 8 to 200 μg/mL, with IC_50_ values higher than 200 μg/mL.

### 4.6. Cytotoxic Activity

Cytotoxicity was evaluated using the survival method with *A. salina* crustaceans. The total extract of the bulbs showed no significant cytotoxicity at any concentration. In contrast, the standard drug actinomycin D exhibited strong cytotoxicity, causing a 96% mortality rate in larvae at the tested concentration of 10 mg/mL.

## 5. Discussion

*Fritillaria karelinii*, known as Sha-Beimu in TCM, has been primarily used for treating respiratory diseases. However, there is limited information regarding its morpho-anatomical and phytochemical properties, with most existing data available in Russian. The novelty of the present work lies in providing a combined morpho-anatomical description, HPTLC fingerprinting, and GC-MS profiling of bulb extracts, alongside biological activity screening, which together establish a more integrative characterization of this species compared to previous reports that focused only on antioxidant potential (26).

The methanol extract of *F. karelinii* was analyzed via GC-MS, revealing the presence of mainly fatty acids as dominant constituents. Although several alkaloid-related signals were detected, the GC-MS method applied was limited in its ability to detect other compound classes, such as phenolics and flavonoids, which are often associated with antimicrobial and antioxidant effects. The identified fatty acids included linoleic acid (40.86%), palmitic acid (30.58%), linolenic acid (13.30%), and stearic acid (5.85%). Given that GC-MS is best suited for volatile and semi-volatile compounds, future analyses should incorporate complementary methods such as LC-MS or HPLC to characterize polar phytochemicals, particularly phenolic compounds and flavonoids (27, 33).

For both alkaloids, the MF and RMF values exceed 870, indicating a high level of agreement between the experimental spectra and the library data. According to the NIST library criteria, MF > 800 and RMF > 850 values correspond to high identification reliability. The first compound, 5α-cevane-3β,20-diol (C_27_H_45_NO_2_), has a theoretical molecular weight of 415.34 Da and is characterized by the presence of two hydroxyl groups at positions 3β and 20. The second compound, 3β,20-dihydroxy-5α-cevan-6-one (C_27_H_43_NO_3_, fritillarine), is distinguished by the presence of a carbonyl group at position C-6, which is confirmed by the observation of a characteristic fragment [M-15]^+^ (m/z 414) and an additional peak at m/z 396, corresponding to water loss. The data obtained confirm that both compounds are typical steroidal alkaloids of plants of the genus *Fritillaria*. High MF and RMF values, as well as characteristic fragmentary decay, confirm the correctness of their identification.

The antioxidant potential of the *F. karelinii* extract was assessed using DPPH and FRAP assays. In the DPPH assay, the total extract exhibited only 30.52% scavenging activity, compared to gallic acid (93.40% inhibition at 1 mg/mL). The FRAP assay indicated a ferric reducing capacity of 1.0578 µg/mL gallic acid/g dry weight for the extract, versus 1.8705 µg/mL for gallic acid. These results clearly show that the extract possessed weak antioxidant activity compared to the positive control. The discrepancy between DPPH and FRAP values also underscores the limited antioxidant potential of the extract and suggests that phenolic compounds, typically responsible for strong antioxidant activity, may be present in very low concentrations or absent (34).

Polyphenols, including flavonoids, phenolic acids, and tannins, are the primary active compounds associated with antimicrobial activity in medicinal plants. However, the methanolic extract and fractions of *F. karelinii* did not exhibit significant antimicrobial effects on the tested microorganisms. While low extract concentration may contribute to this outcome, another plausible explanation is the absence or very low abundance of polyphenolic compounds in the bulbs. This highlights a limitation of the current experimental design, suggesting that testing a wider range of concentrations, fractionated extracts, and different solvents is necessary to fully evaluate the antimicrobial potential (35-37).

The use of *A. salina* crustaceans in acute toxicity assays is highly valuable due to its simplicity and reproducibility. In our study, the methanolic extract of *F. karelinii* did not show any significant cytotoxic effects at the tested concentrations, with only 4% mortality observed in larvae. In contrast, actinomycin D caused 63 - 96% mortality. While this suggests that the extract lacks acute toxicity under these conditions, the result should be interpreted cautiously. The absence of cytotoxicity may reflect either a true lack of toxic constituents or the limitations of the extraction method and concentration range applied. Broader cytotoxicity assays, including mammalian cell lines, would be necessary to more accurately determine potential biological safety or activity (38, 39).

## Data Availability

The dataset presented in the study is available on request from the corresponding author during submission or after publication.
